# Surviving Critical Low Hemoglobin Levels and Pica

**DOI:** 10.7759/cureus.38812

**Published:** 2023-05-10

**Authors:** Syeda Juwairiyyah Fatima, Nisha Nepal, Daniel Ochieta

**Affiliations:** 1 Hematology, University of Vermont, Burlington, USA; 2 Internal Medicine, Danbury Hospital, Danbury, USA; 3 Hematology, Danbury Hospital, Danbury, USA

**Keywords:** uterine leiomyoma, lowest hemoglobin that is life-compatible, uterine mass, severe pica, severe iron deficiency, critical low hemoglobin

## Abstract

Iron deficiency is the most common nutritional deficiency. Pica is commonly associated with iron deficiency anemia (IDA). A case of a 40-year-old female who presented with a critical record of low hemoglobin (Hgb) (1.6 g/dL) with severe iron deficiency and pica with no lasting deficits despite such low hemoglobin is discussed in this article. The patient presented to the emergency room with complaints of weight loss, weakness, palpitation, fatigue, dysphagia, and on-and-off vomiting for about a year and severe menorrhagia for about one and a half years. She also has had pica for the past several years where she eats and chews toilet paper. Several of her female family members also have pica. She was found to have critically low hemoglobin of 1.6 g/dL and serum iron of 8 ug/dL and ferritin of less than 1 ng/mL. The patient was treated with six units of packed red blood cells and IV and oral iron supplementation. She was discharged with a hemoglobin of 7.3 g/dL. She was later found to have a 9.6 cm uterine mass that is consistent with leiomyoma (fibroid) in transvaginal ultrasound and is following up with a gynecologist for the definitive management. She did not have lasting deficits from the critically low hemoglobin and has stopped engaging in pica behavior.

## Introduction

Iron deficiency is the most common nutritional deficiency in the world affecting up to 25% of the population [1]. An unusual link exists between iron deficiency and pica, as 'noted in numerous studies [2]. The American Psychiatric Association’s Diagnostic and Statistical Manual of Mental Disorders, Fifth Edition (DSM-5) defines pica as persistent eating of nonnutritive, nonfood substances for at least one month. The condition is seen in 10%-15% of individuals with learning disabilities, 20% of pregnant women, and 25%-33% of young children [3]. Iron deficiency anemia (IDA) makes up half of all global cases of anemia [4]. This case report will discuss a patient with severe iron deficiency anemia with critically low hemoglobin (Hgb) and severe pica, which did not impose her with long-lasting deficits.

## Case presentation

A 40-year-old African American female with no prior medical history presented to the emergency room for weight loss, weakness, palpitations, fatigue, dyspnea, dysphagia, and on-and-off vomiting. The patient attributed many of her symptoms to a COVID-19 infection she had a year and a half ago, including menorrhagia. She has been having heavy menstrual bleeding for one and a half years, which she attributes to the COVID-19 infection she had 1.5 years ago. Her menses lasts up to seven days with the first three days being the heaviest. She reports needing to change pads every 20 minutes and bleeds through to her clothing. She uses a towel at night because she soaks through to her bedding. The patient also reported that she had pica. She began eating toilet paper several years ago. It takes her one week to work her way through one roll of toilet paper. Interestingly, the patient reported that several of her female family members also have pica. Her mother, sister, and niece have all eaten toilet paper during their lifetime. The patient reported no known personal or family history of hematological disorders including anemia and thalassemia.

**Figure 1:**
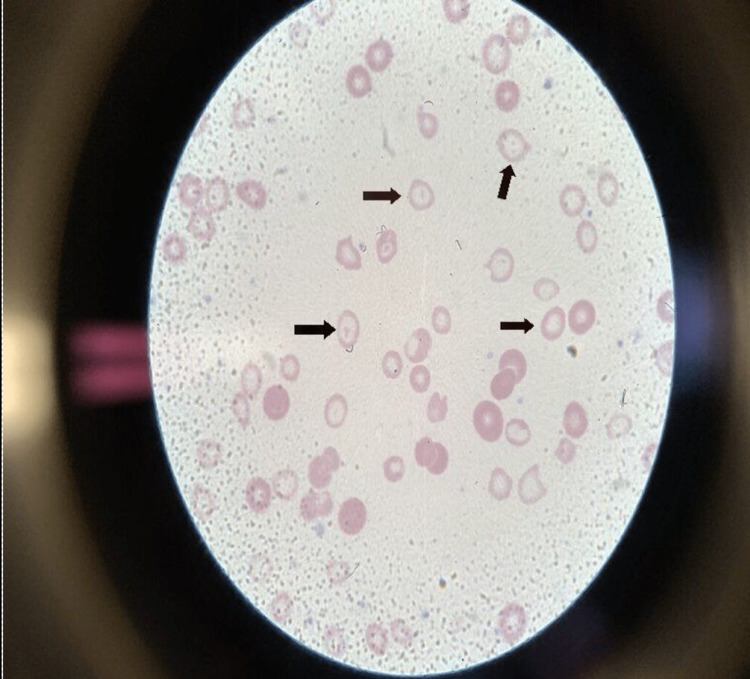
Patient’s peripheral blood smear with arrows pointing at some of the numerous hypochromic cells present

On presentation, the patient was afebrile, with a heart rate of 84 beats/minute, blood pressure of 152/82, respiratory rate of 16 breaths/minute, and O2 saturation of 100%. On examination, the patient looked pale and had koilonychia and a flow murmur best heard on the left sternal border. Chemistry was significant for bicarbonate of 21 mmol/L and creatinine of 1.14 mg/dL. The patient’s CBC was significant for a hemoglobin of 1.6 g/dL, hematocrit (Hct) of 6.9%, mean corpuscular volume (MCV) of 57.5 fL, reticulocyte count of 0.8%, and reticulocyte production index of 0.1. The patient’s peripheral smear had marked anisopoikilocytosis with microcytic hypochromic red blood cells, target cells, teardrop cells, a few helmet-like folded cells, reticulocytes, and nonspecific poikilocytoses with differential diagnosis of iron deficiency anemia versus hemoglobinopathy (Figure 1). Follow-up iron studies were significant for serum iron of 8 ug/dL, total iron-binding capacity (TIBC) of 465 ug/dL, transferrin saturation of 2%, and ferritin of less than 1 ng/mL. The patient also had a negative stool guaiac test. Her vitamin B12 and folate levels were normal.

The patient was admitted for management of severe symptomatic iron deficiency anemia (IDA) of unknown etiology. Given the marked anemia at a Hgb of 1.6 g/dL, she received a total of six units of packed red blood cells. The patient received two doses of IV ferric gluconate and oral ferrous sulfate. She reported marked improvement in her symptoms, especially the fatigue she was experiencing prior to admission. The patient was discharged with a Hgb of 7.3 g/dL and a Hct of 23.9%.

Further evaluation with hemoglobin electrophoresis showed no evidence of abnormal hemoglobin or beta thalassemia, although alpha thalassemia could not be excluded without further testing. However, the sample for electrophoresis was taken after the patient received a few units of blood, so the results could be skewed.

Our patient’s severe IDA could be in the setting of menorrhagia. However, thalassemia and gastrointestinal (GI) bleeding have to be ruled out as well. The patient was discharged with instructions to follow up with gastroenterology for an upper and lower endoscopy to evaluate for possible Plummer-Vinson syndrome, especially because of her symptom of dysphagia, and to rule out other causes of GI bleeding. She was also recommended to see hematology for anemia follow-up and repeat electrophoresis in 3-4 months, cardiology to follow-up on possible left ventricular hypertrophy in the setting of severe anemia [5], and gynecology for further workup of menorrhagia, which could be a cause of her severe IDA. After being discharged, she had a transvaginal ultrasound and was found to have a 9.6-cm uterine mass that is consistent with leiomyoma (fibroid), but on initial diagnosis of a solitary large presumed fibroid, the possibility of leiomyosarcoma cannot be excluded. She is yet to follow up with the gynecologist for definitive management. She also followed up with the cardiologist, and an echocardiogram was done, which showed left ventricle mild hypertrophy with a 60%-65% ejection fraction.

## Discussion

This patient presented with a hemoglobin of 1.6 g/dL, one of the lowest levels reported in the literature. Some of the lowest recorded hemoglobin levels in non-trauma patients are relevant for a 21-year-old male with a hemoglobin of 1.2 g/dL in the setting of paroxysmal nocturnal hemoglobinuria and a 44-year-old female with a hemoglobin of 1.3 g/dL secondary to uterine fibroid bleeding [6,7]. Both of those patients presented with lactic acidosis secondary to severe anemia. The patient discussed in this case report had insignificant laboratory results apart from iron deficiency anemia. A further review of the literature for patients with hemoglobin values in a similar range to this patient is relevant for a 97-year-old male with a hemoglobin of 1.7 g/dL in the context of a chronic gastrointestinal bleed [8] and a 29-year-old female with a hemoglobin of 1.7 g/dL in the setting of celiac disease [9].

To the best of our knowledge, a previous case of concurrent severe iron deficiency anemia with hemoglobin as low as 1.6 g/dL and pica has never before been documented in the literature. This patient provides interesting insight into the well-documented yet poorly understood relationship between pica and iron deficiency anemia. A case series of three patients indicated the resolution of pica with iron supplementation [10]. On gastroenterology follow-up, our patient also reported a resolution of pica, perhaps due to improvement in her anemia secondary to iron supplementation with oral ferrous sulfate. Although this patient’s hemoglobin electrophoresis did not indicate hemoglobinopathies, pica has been noted to be correlated with lower hemoglobin levels in pediatric patients with sickle cell disease [11]. Another study of blood donors demonstrated a significant probability of iron deficiency in patients who self-reported pica behavior [12]. Moreover, given the patient’s family history of pica in many of her female relatives, it is possible that there is an underlying genetic component contributing to her anemia-like thalassemia. There is a hypothesis that the consumption of earth substances serves as a compensatory mechanism for individuals experiencing deficiencies in iron, zinc, or calcium. As a result, those with the highest requirements for these essential nutrients are more likely to engage in geophagy practices [13].

## Conclusions

In conclusion, this case report discusses one of the lowest presenting hemoglobin values in a hemodynamically stable patient ever discussed in the literature. There are few people who can still maintain their day-to-day activities even with hemoglobin of less than 2 g/dL. Since admission, the patient has recovered from her severe symptomatic anemia and has stopped engaging in pica behavior. She has no lasting deficits from the severe anemia and continues to receive outpatient workups for possible causes of the iron deficiency anemia, including workup and management of her uterine mass, possible upper and lower endoscopy to rule out gastrointestinal bleeding, and a repeat hemoglobin electrophoresis.

## References

[1] Coad J, Conlon C (2011). Iron deficiency in women: assessment, causes and consequences. Curr Opin Clin Nutr Metab Care.

[2] Barton JC, Barton JC, Bertoli LF (2010). Pica associated with iron deficiency or depletion: clinical and laboratory correlates in 262 non-pregnant adult outpatients. BMC Blood Disord.

[3] Barker D (2005). Tooth wear as a result of pica. Br Dent J.

[4] Camaschella C (2019). Iron deficiency. Blood.

[5] Park SK, Jung JY, Kang JG, Hong HP, Oh CM (2020). Association of Left Ventricular Hypertrophy with Hemoglobin Levels in Nonanemic and Anemic Populations. Cardiology.

[6] Essex DW, Jin DK, Bradley TP (1997). Lactic acidosis secondary to severe anemia in a patient with paroxysmal nocturnal hemoglobinuria. Am J Hematol.

[7] Ntalianis A, Mandrekas K, Papamichael C, Anastasiou-Nana MI (2009). Life-threatening iron deficiency anemia and profound lactic acidosis due to uterine fibroid bleeding. Am J Emerg Med.

[8] Jost PJ, Stengel SM, Huber W, Sarbia M, Peschel C, Duyster J (2005). Very severe iron-deficiency anemia in a patient with celiac disease and bulimia nervosa: a case report. Int J Hematol.

[9] Kyvetos A, Panayiotou S, Voukelatou P, Vrettos I, Boulmetis G (2022). The case report of a 97-year-old patient with chronic anemia and hemoglobin value of 1.7 g/dl and review of the literature. Cureus.

[10] Khan Y, Tisman G (2010). Pica in iron deficiency: a case series. J Med Case Rep.

[11] Ivascu NS, Sarnaik S, McCrae J, Whitten-Shurney W, Thomas R, Bond S (2001). Characterization of pica prevalence among patients with sickle cell disease. Arch Pediatr Adolesc Med.

[12] Bryant BJ, Yau YY, Arceo SM, Hopkins JA, Leitman SF (2013). Ascertainment of iron deficiency and depletion in blood donors through screening questions for pica and restless legs syndrome. Transfusion.

[13] Ganesan PR, Vasauskas AA (2023). The association between pica and iron-deficiency anemia: a scoping review. Cureus.

